# On the Use of the Muriate of Cocaine in Diseases of the Throat

**Published:** 1886-05-20

**Authors:** P. R. Cortelyon

**Affiliations:** Marietta, Ga.


### Southern Medical Record.

A MONTHLY JOURNAL OF PRACTICAL MEDICINE.

☞All Communications must be addressed to R. C. WORD, M. D., Managing Editor.

"*Quicquid Præcipies Esto Brevis.*"

VOL. XVI. ATLANTA, GA., MAY 20, 1886. No. 5.

### ORIGINAL AND SELECTED ARTICLES.

### ON THE USE OF MURIATE OF COCAINE IN DISEASES OF THE THROAT.

BY P. R. CORTELYON, M. D., MARIETTA, GA.

Thinking that, perhaps, some of the many readers of your excellent journal might be interested in reading a report of the results following the use of cocaine solution in the treatment of some diseases of the throat, I send you a short history of a few cases.

Mr. ——, patient, came under my care early in January, 1885, complaining of cough, pain in the throat, and inability to speak above a hoarse whisper. On examination, I discovered marked evidence of tubercular deposit in right lung, with softening and a general catarrhal condition of the whole lung. The laryngescope revealed a congested and puffy condition of the supra-arytænoid cartilages, and general pharyngitis. I used various local measures, with brush and spray, without giving much relief. I then made a daily application of 4-per cent. solution of muriate of cocaine to supra-arytænoid cartilage and pharynx with brush. The patient expressed great satisfaction at the comfort given by the medicine, as it relieved his pain and checked the cough. Two weeks' treatment caused the aphonia to disappear, and the general distress about the throat was relieved for several weeks, although the disease of the lungs progressed and finally proved fatal.

CASE II.—Mr. W—— consulted me early in February, 1885, suffering with pain in throat; had cough, and inability to speak above a whisper. Physical examination revealed marked evidence of phthisis pulmonalis in third stage, with ulcerated condition of vocal cords. Used spray of bromide of potass, ʒ ss ; aquæ, ℥ i ; then applied cocaine solution freely to vocal cords and pharynx. This treatment was continued daily for some days, giving great comfort to the patient, but, of course, with no arrest of the disease, which eventually proved fatal.

CASE III.—Mr. B—— consulted me in August, 1885, for throat trouble. Examination revealed chronic catarrhal pharyngitis. Applied argenti nitras, ʒ i; water, ℥ i, to parts with camel's hair brush, every third day, and ordered gargle, acidi salicylic, ʒ i ; listerini, ℥ i ; glycerini, ℥ i ; aquæ, ℥ ii. September 26, patient complained of pain and irritated condition of throat. Applied 4-per cent. solution of cocaine, which gave relief. Repeated application daily for several days, when he felt so much relieved that he discontinued treatment.

CASE IV.—Mr. K—— consulted me September 24, 1885, complaining of sore throat, and hacking cough, preventing sleep. On examination, discovered some consolidation of left lung at apex ; throat congested and irritable. Applied "cocaine solution" daily, giving ease, and relieving cough. After several days he was so much benefitted that he did not feel the need of cocaine. Several times since then, when throat has become sensitive and irritable, 4-per cent. solution of cocaine has given relief.

CASE V.—Miss G—— was brought to me October 2, 1885, by the physician who had been treating her for throat disease for some time, by means of gargles and local applications of tannic acid and glycerine. Examination with laryngoscope showed marked congestion of the mucous membrane of the supra-arytænoid cartilages and of the lingual sinus on right side. She complained of much pain in the throat, and difficulty in deglutition. Made application with camel's-hair brush of 4-per cent. solution of cocaine, which gave immediate relief to pain, and made her throat comfortable for twenty-four hours. After a week's treatment, patient expressed herself free from all pain, and stopped treatment.

CASE VI.—Mrs. T—— consulted me November 21, 1885 ; suffering with severe pain in throat, and cough, night sweats, and general debility ; would not have lungs examined. Used twice daily spray of potass bromide, ʒ ss ; aquæ, ℥ i, to throat, and daily applications of cocaine solution. Ordered tonics—cod-liver oil and acid sulph. aromatic—for night sweats. She improved very rapidly, and expressed the greatest satisfaction on the great benefit of the throat treatment, as it relieved pain and cough, and so enabled her to rest at night, and eat her meals without distress. After one month's treatment her throat felt so well, and her general condition was so much improved, that she discontinued treatment ; and since that time she has kept well and free from trouble.

The beneficial results following the use of cocaine in these few cases prove that in it we have a valuable medicine in the treatment of throat troubles ; and while it may not of itself be curative, certainly allays irritation and pain, and thus enables Nature to accomplish the cure.

